# The importance of sharing global forest data in a world of crises

**DOI:** 10.1038/s41597-020-00766-x

**Published:** 2020-12-01

**Authors:** Jingjing Liang, Javier G. P. Gamarra

**Affiliations:** 1 Forest Advanced Computing and Artificial Intelligence Laboratory, Department of Forestry and Natural Resources, Purdue University, West Lafayette, USA; 2 National Forest Monitoring (NFM) Team, Forestry Division, Food and Agriculture Organization of the United Nations, Rome, Italy

**Keywords:** Forestry, Ecology

## Abstract

Open access to global forest data, especially ground-measured (*in situ*) records, is critical for saving the world’s forest systems. Integrated approaches to achieve sustainable data openness will involve legal assurances, shared ethics, innovative funding schemes and capacity development.

Amid ongoing global crises related to climate change, environmental degradation, pandemics, poverty, peace and justice, the international community has established 17 Sustainable Development Goals (SDG) as a roadmap to navigate the humanity through these uncharted waters. In parallel, signatories of the Paris agreement are committed to climate action through their Nationally Determined Contributions^1^. Although SDGs and Paris agreement pledges are complementary, the common roadmap of implementation is underperforming. New, unexpected global crises emerge almost every year from a lack of stewardship to stem the biosphere’s degradation^2^.

Forest ecosystems are a critical node in an integrated systems approach to tackling global crises and achieving SDGs. Specifically, forest ecosystems provide crucial services such as absorbing 30% of anthropogenic greenhouse gas emissions^3^, producing wood fuel or charcoal to 2.4 billion people^4^, and being the most important global repository of terrestrial biodiversity, with 10% of its global area dedicated to biodiversity conservation^5^. Forest ecosystems sustain food, water and energy security, and human well-being^6^, but deforestation and forest degradation are destroying a massive amount of forests worldwide, rendering more than 40,000 tropical tree species at risk^7^. Central to saving the world’s forest ecosystems, forest data enable the quantification of deforestation and forest degradation^5^, and facilitate research into effective protection and conservation measures.

Open access policies, as well as the development of supportive cyberinfrastructures, are key to meet an increasing public demand for forest information^8^ for research, monitoring, policy-making, and other purposes. Over the past decades, users of forest data have expanded from a small group of authorized forestry researchers, government officials and private sector experts to all in the public domain who can benefit from this information. These data are now used by citizen scientists, environmentalists, and researchers from disciplines such as ecology, Earth sciences, and biological conservation. The quantity and quality of open forest data, however, still remains limited, especially in the tropics and other regions of the Global South (Fig. 1).

**Fig. 1:**
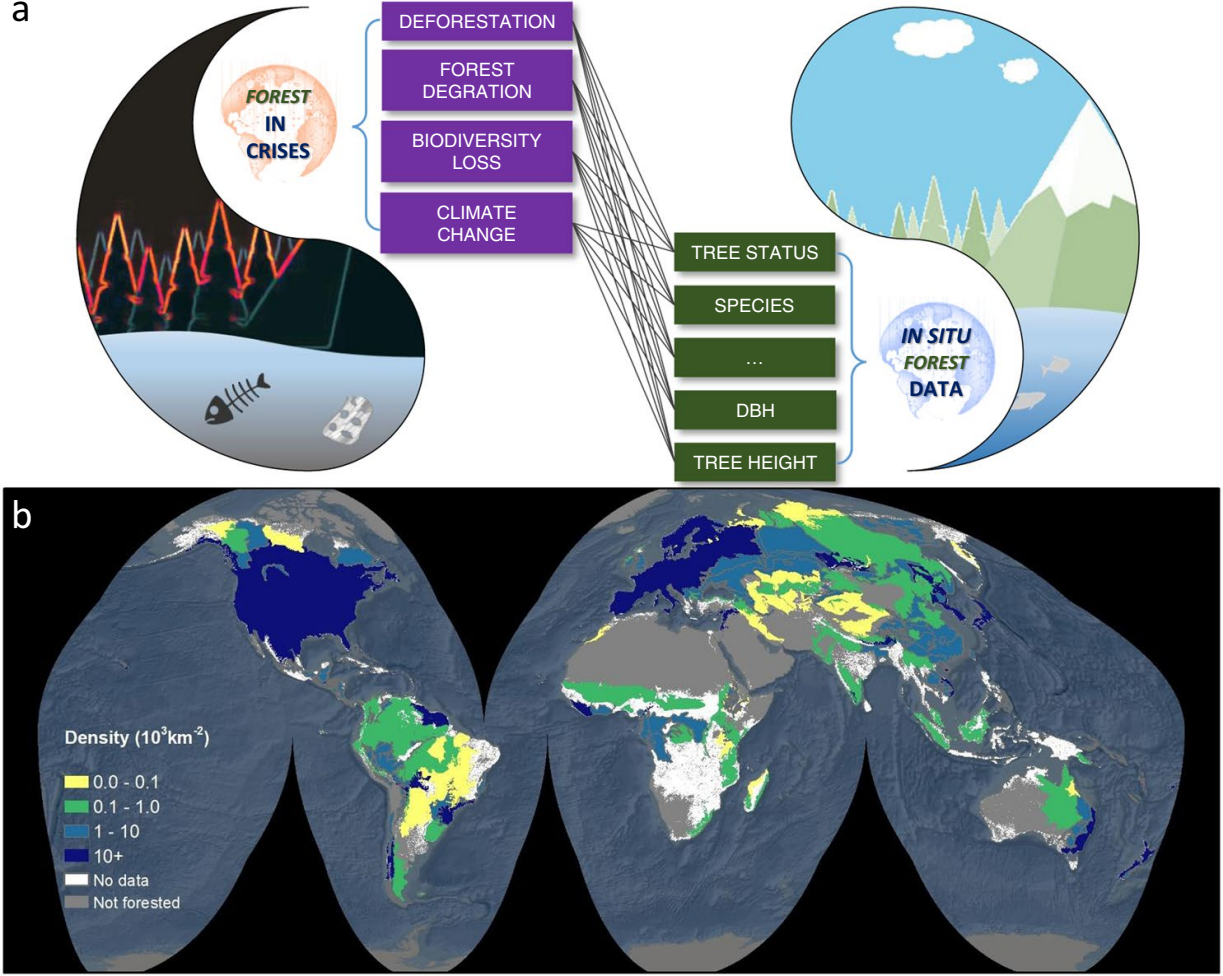
Open *in situ* forest data are critical for the monitoring and mitigation of deforestation, forest degradation, biodiversity loss, and climate change (**a**), but sampling density of *in situ* forest data across the world, in number of sample plots per 1000 square kilometers, is generally low (**b**). Sampling density was calculated at the ecoregion^25^ level, based a global database compiled by the Global Forest Biodiversity Initiative (GFBI, https://www.gfbinitiative.org/). Containing approx. 1.3 million sample plots, GFBI is one of the largest *in situ* forest datasets.

Forest data represent information systematically collected via remote sensing and ground (*in situ*) measurements for quantifying the status of a forest, including its ecological and economics conditions. While data from government-owned satellites and other remote sensing observations are increasingly being openly shared^9^, the sharing of *in situ* forest data is still very limited. *In situ* forest data, similar to many other land-based sector data, originate from area-based sampling frame surveys. Three attributes are commonly measured and recorded in most *in situ* forest data—tree status (e.g. live, dead, recruitment, etc.), species, and diameter at breast height (dbh). Sometimes, other attributes can be taken at the same time, such as tree height and timber quality. Based on these elemental attributes, one can derive basal area, species diversity, site index, stand volume, and other metrics to quantify the status of a forest. Due to limitations in budget and labor, *in situ* forest data typically present relatively low sampling intensities over loosely defined tree target populations that are *a priori* unquantifiable. For example, regions with the highest sampling densities in the world encompass around three sample plots per one square kilometer of forested area, while for most of the world’s forested areas, the sampling density is less than one plot per 100 square kilometers (Fig. 1b).

Here, we discuss the importance of sharing global forest data, especially *in situ* records, and outline major obstacles to sharing these data, as well as ongoing efforts and potential solutions to overcome them.

## Forest Ecosystems in Crisis

Climate change, biodiversity loss, as well as deforestation and forest degradation, interconnected through potential positive feedbacks (e.g.^10^), are the three major crises collectively affecting forests worldwide.

Climate influences the structure and function of forest ecosystems and plays an essential role in forest health. A changing climate may worsen many of the threats to forests, such as extreme weather damage, pest outbreaks, fires, human development, and drought^11^. A changing climate directly and indirectly affects the growth and productivity of forests, and alters the growing conditions, rendering some local tree species at risk if conditions in their current geographic ranges are no longer suitable. Meanwhile, climate change is found to increase the risk of drought in some areas and the risk of extreme precipitation and flooding in others^12^. The combined drought and extreme weather events increase wildfire risk, and hamper trees’ ability to protect themselves from insect pests.

Under climate change, our planet’s biodiversity is undergoing an accelerated loss. From tropical rainforest to arctic tundra, substantial biodiversity loss at a global scale is undermining global terrestrial ecosystems at an alarming rate^6^. The role of biodiversity in the functioning of forest ecosystems has been under intensive investigation for the past two decades, and mounting evidence has shown that forest ecosystem function and productivity is affected by the loss of biodiversity^13^. To this end, biodiversity conservation is considered at least as important to achieving SDGs as the efforts to mitigate global climate change^6^, and the two missions are closely connected and complementary. Ecosystems with greater biodiversity are better equipped to cope with climate change, extreme weather events and the emergence of diseases. On the other hand, climate change mitigation, along with a reduction in over-exploitation of resources, habitat destruction, pollution and the spread of invasive species, is one of the most effective measures to conserve and promote biodiversity^6^.

Deforestation and forest degradation—including land use change and forest fragmentation—are tightly linked to climate change and biodiversity loss, and increase the risks of infectious diseases^14^. Human settlements in degraded forests bring humans into close contact with new opportunistic animals, and animals that struggle to stay in isolated fragments of natural vegetation. This has increased direct or indirect exposure of humans to zoonoses—yellow fever, ebola and coronavirus among others—which often spill over from one species to another at the margins of forests^14^. In fragmented forests and associated forest edges, agricultural fields, and pastures, many wildlife species are likely to disappear as forests are cleared, while others that have been able to adapt may become more concentrated, further increasing the rate of infections. The protection of forests, particularly in the tropics, and conservation of wildlife are therefore important to keep infectious agents in check.

## Importance of Forest Data

Since the advent of remote sensing technology, some forest attributes can be measured using airborne, spaceborne and ground-based sensors, but *in situ* data still remain an indispensable and irreplaceable source of information especially for quantifying biodiversity and ground-truthing remote sensing data. For instance, with existing remote sensing and image processing technologies, it is difficult to identify tree species in a mixed-species forest with precision^15^, especially for tropical forests and species-rich temperate forests. To this end, *in situ* data remain the most reliable information for forest resources and tree biodiversity assessment and monitoring, and will likely remain so in the foreseeable future^16^. Furthermore, due to a lack of *in situ* data for ground-truthing, the accuracy of many remotely sensed map products remains unassessed^17^, jeopardizing their applications in timber and carbon accounting, biodiversity research and conservation.

Assessing and monitoring biodiversity—central to global biodiversity conservation—is restricted by data limitation^18^. To date, large-scale conservation efforts have relied heavily on presence-only species count information (e.g.^19^) inferred from expert opinions or sparse incidence data collected from *ad hoc* local studies^20^. As a result, species abundance information for the majority of forest communities across the world has been relatively scarce, despite the importance of such information for prioritizing biological conservation^21^. Assessing and monitoring biodiversity across the world’s forest ecosystems will provide much needed guidance for future forest conservation efforts and considerably improve our understanding of the drivers of biodiversity loss under global environmental change.

Degradation of forest ecosystems is manifested in many different ways, including a reduction in their ecological properties (such as receded canopy cover and reduced biodiversity) and ecosystem services (such as lowered timber productivity/quality and impaired carbon sequestration capability), as well as a decline in their resilience to and recovery from disturbances^22^. In practice, the United Nations Framework Convention on Climate Change (UNFCCC), following recommendations from the Intergovernmental Panel on Climate Change (IPCC) strongly advises combined approaches of *in situ* inventory and remote sensing data when assessing forest degradation and associated changes in forest area and carbon stocks^23^.

## Sharing of *in Situ* Forest Data

The last 10 years have witnessed a substantial increase in transparent and credible sharing of *in situ* forest data, which has resulted in an upsurge in forest research (e.g.^3,7,13^) and monitoring^16^ at a global scale. The amount of shared *in situ* forest data, however, does not seem to meet the urgency of our global crises. The Global Forest Biodiversity Initiative (GFBI) database is one of the largest *in situ* forest databases consisting of 1.3 million sample plots across the world. Based on this, the sampling density of *in situ* forest data is commonly more than 10 plots per 1,000 square km in developed countries, whereas the sampling density for the tropics and other regions of the Global South is largely less than one plot per 1,000 square km (Fig. 1). Such a stark difference—between one and three orders of magnitude, depending on the region—in forest data availability across various biomes poses a major issue for tropical regions which host the vast majority of biodiversity. Overall, less than 30% of *in situ* forest datasets are open access.

A lack of motivation constitutes one of the greatest hurdles in open sharing of *in situ* forest data. Except for a few countries mandated by their domestic law to share the national forest inventory (NFI) data, most of the remaining countries are reluctant or sometimes opposed to open sharing of forest data. Reasons for this include institutional miscoordination, national security, privacy, and potential disadvantages in the international negotiation on climate change offsets^24^. A lack of government transparency in this area can result in corruption, misinformed decision-making, and difficulties in the enforcement of forest protection and conservation. Moreover, a lack of motivation to share data also applies to many non-government data owners, who are not motivated to share these data without clear benefits, because *in situ* forest inventory data are often associated with high logistic and labor costs, especially in the tropics and other regions of the Global South.

Major efforts have been made to overcome this hurdle in open sharing of forest data. The Food and Agriculture Organization of the United Nations (FAO) has initiated an open data initiative as part of a project to increase transparency in the forest sector (CBIT-Forest, http://www.fao.org/in-action/boosting-transparency-forest-data/en/). The FAO recognizes both the national contexts of many countries and the increasing demands of international donors that support the development of NFIs in recipient countries. It aims to capitalize on its historical presence in these countries and on the recent growth in NFIs, many of which it directly supports. It will offer countries open data platforms and analysis tools, while helping to provide forest scientists worldwide with vast amounts of much-needed forest microdata from mostly tropical countries. Simultaneously, tremendous efforts have been made by non-government actors to compile *in situ* forest data and make them available to data users, including the Global Forest Biodiversity Initiative (GFBI), the Amazon Forest Inventory Network (RAINFOR), Global Biodiversity Information Facility (GBIF), the African Tropical Rainforest Observation Network (AfriTRON), and several other international research teams, networks, and initiatives.

## Immediate Actions Needed

The open sharing of *in situ* forest data is directly affecting the public awareness, research, monitoring and policy making of forest resources worldwide. Consequently, our ability to mitigate climate change, biodiversity loss, as well as deforestation and forest degradation largely depends on it. While no single model has been successful in achieving the goal of open *in situ* forest data worldwide, an integration of top-down (such as FAO’s CBIT-Forest transparency initiative) and bottom-up (such as AfriTRON, GBIF, GFBI, RAINFOR) approaches has shown the most promising outcomes. To this end, a shared responsibility by governments, scientists, NGOs, indigenous people, and other forest monitoring groups, in both rich and poor nations, will be key to the success and sustainability of the open sharing of global forest data. Low-income countries in particular need supporting assurances with regard to data governance and sustainability. Clear legal alignments between institutions on data sharing agreements and licenses for redistribution, as well as alignments with open data international standards will provide a solid basis for data governance. Meanwhile, innovative funding mechanisms are needed to promote data sustainability for low-income countries. For example, forest inventories funded for reducing emissions from deforestation and forest degradation, and for promoting conservation, sustainable management of forests and enhancement of forest carbon stocks in developing countries (REDD + ), can be incorporated into existing forest monitoring systems. This type of innovative funding mechanisms - based on shared responsibility - will be critical to sustain the procurement of *in situ* forest data from low-income countries. This includes multiple purposes such as the management of forest resources, the mitigation of climate change, and the conservation of biodiversity. The severe shortage of experts and facilities poses another major hurdle for the collection of forest inventory data, especially in the tropics and other regions of the Global South where sampling density of *in situ* forest data is very low (Fig. 1). Proper education and capacity development of new generations of tree taxonomists, forest scientists and foresters will be critical to improve forest data coverage, while bringing tangible benefits to the local economy of rural communities around the world. Nevertheless, the urgency to fulfill the SDGs and protect forest ecosystems requires immediate worldwide attention to open forest data.
